# ISVASE: identification of sequence variant associated with splicing event using RNA-seq data

**DOI:** 10.1186/s12859-017-1732-7

**Published:** 2017-06-28

**Authors:** Hasan Awad Aljohi, Wanfei Liu, Qiang Lin, Jun Yu, Songnian Hu

**Affiliations:** 1 Joint Center for Genomics Research (JCGR), King Abdulaziz City for Science and Technology and Chinese Academy of Sciences, Prince Turki Road, Riyadh, 11442 Saudi Arabia; 2 CAS Key Laboratory of Genome Sciences and Information, Beijing Institute of Genomics, Chinese Academy of Sciences, NO.1 Beichen West Road, Chaoyang District, Beijing, 100101 China; 3 Current address: Grail Scientific Co. Ltd., Room 26–1, Build A, Meilong Jiayuan, NO. 80 South Nanjing Street, Heping District, Shenyang, Liaoning 110000 China

**Keywords:** Sequence variant, Splicing event, Association, RNA-seq, DNA mutation, RNA editing

## Abstract

**Background:**

Exon recognition and splicing precisely and efficiently by spliceosome is the key to generate mature mRNAs. About one third or a half of disease-related mutations affect RNA splicing. Software PVAAS has been developed to identify variants associated with aberrant splicing by directly using RNA-seq data. However, it bases on the assumption that annotated splicing site is normal splicing, which is not true in fact.

**Results:**

We develop the ISVASE, a tool for specifically identifying sequence variants associated with splicing events (SVASE) by using RNA-seq data. Comparing with PVAAS, our tool has several advantages, such as multi-pass stringent rule-dependent filters and statistical filters, only using split-reads, independent sequence variant identification in each part of splicing (junction), sequence variant detection for both of known and novel splicing event, additional exon-exon junction shift event detection if known splicing events provided, splicing signal evaluation, known DNA mutation and/or RNA editing data supported, higher precision and consistency, and short running time. Using a realistic RNA-seq dataset, we performed a case study to illustrate the functionality and effectiveness of our method. Moreover, the output of SVASEs can be used for downstream analysis such as splicing regulatory element study and sequence variant functional analysis.

**Conclusions:**

ISVASE is useful for researchers interested in sequence variants (DNA mutation and/or RNA editing) associated with splicing events. The package is freely available at https://sourceforge.net/projects/isvase/.

**Electronic supplementary material:**

The online version of this article (doi:10.1186/s12859-017-1732-7) contains supplementary material, which is available to authorized users.

## Background

Alternative splicing is a normal phenomenon in eukaryotes and greatly increase the biodiversity of proteins. About 95% of multi-exonic genes are alternatively spliced in human [1]. The extreme example is the *Drosophila Dscam* gene, which produces thousands of protein isoforms by alternative splicing [2]. Classic pre-mRNA splicing is recognized and regulated by core splicing signals (5′ splice site (5′ ss), 3′ splice site (3′ ss), branch point sequence) and auxiliary sequences (splicing regulatory elements). Aberrant RNA splicing has become a common disease-causing mechanism, which can lead to hereditary disorders and cancers. Recent studies indicate that one third or a half of disease-causing mutations can affect RNA splicing [3, 4]. Therefore, identification of sequence variant associated with splicing event (SVASE) becomes a meaningful procedure to illustrate the pathogenesis of diseases. Usually, sequence variant can result in aberrant splicing by disturbing regulatory element sequence or changing splice site [5]. For example, two sequence variants in splicing regulatory elements induce the aberrant splicing of *BRCA2* exon 7 [6]. Moreover, RNA editing also can effect RNA splicing in transcriptome level [7].

Nowadays, RNA-seq has become a routine method for gene expression calling in multiple studies and can be also used to identify sequence variant and splicing event simultaneously [8, 9]. However, there is only one bioinformatic tool (PVAAS) available for directly identifying genome-wide SVASE [10], which has some shortages, such as dependency on known splicing sites, only for novel splicing events, high false positive and long running time. Herein, we develop ISVASE, a suite of Perl scripts, to address the shortcomings of PVAAS and provide new functions for downstream analysis. The only necessary input files are genome sequence (FASTA format) and sequence alignment (BAM or SAM format) [11] files. The sequence alignment file must contain split-reads mapping result produced by software like GSNAP [12] and TopHat [13]. We also recommend users to provide known splicing events in GTF, GFF or BED format for junction shift event identification if concerned.

## Implementation

**Fig. 1:**
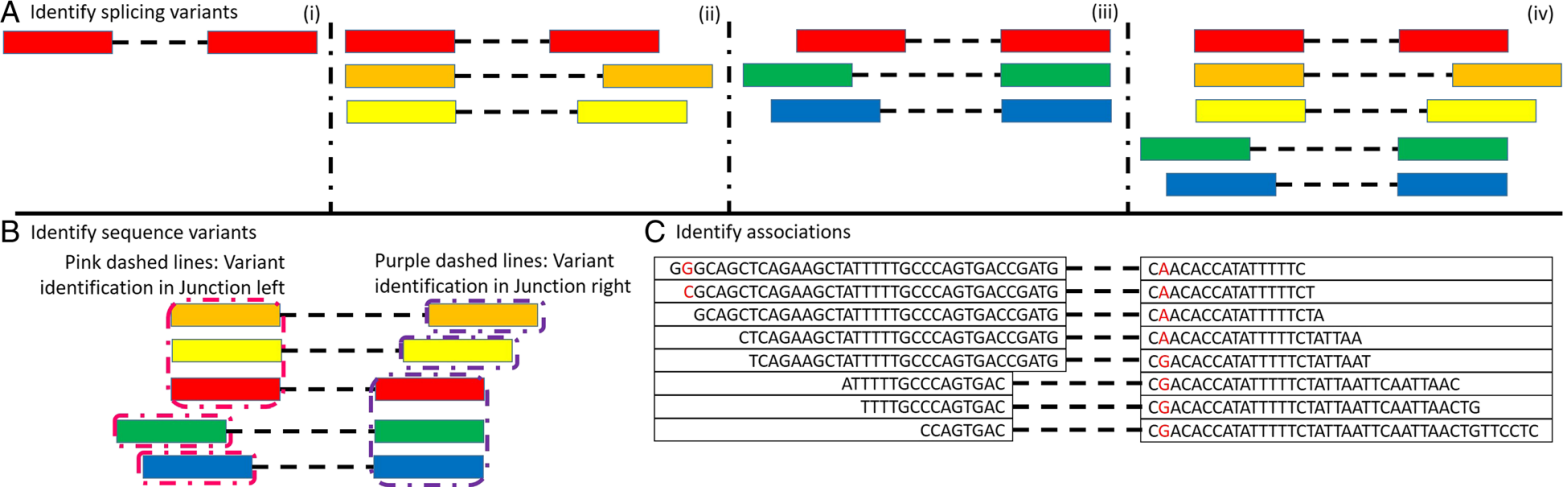
Schematic diagram of the ISVASE software. **a** Identify splicing variants in RNA-seq data. All splicing variants can be divided into four types according to relationship between target splicing variant (*red colour*) and other splicing variants (from left to right): (i) unique splicing variant; (ii) splicing variants with same junction start; (iii) splicing variants with same junction end; and (iv) splicing variants with same junction start or end. **b** Identify sequence variants for each splicing variant and all related splicing variants. To handle all splicing variant types, we identify sequence variants for two parts of splicing separately. In the left part, for junctions with *orange*, *yellow* and *red colour*, the all related splicing variants should be three (all these junctions); however, for junctions with green and blue colour, the total junction is one (itself). Similarly, in the right part, junctions with *red*, *green* and *blue* colour have three all related splicing variants while junctions with orange and yellow colour only has one related junction (itself). **c** Identify associations. This step includes three significant judgements for sequence variants, junction existence and association between sequence variants and junctions, respectively. The example shown two junctions with same junction end. For junction one (top), two sequence variants are identified (left G(ref)- > C(alt) and right G(ref)- > A(alt)). In sequence variant significant judgement, left is filtered (*p* value = 1) while right passes the test (*p* value = 0.0476). In junction significant judgement and association judgement, *p* value of top junction is 0.0128 (significant) and 0.0070 (significant) respectively. Dashed lines represent gaps in the alignment

The basic working principle of SVASE identification includes three main steps: (1) identify alternative splicing events; (2) identify sequence variants in specific splicing event using split-reads; and (3) evaluate the association between sequence variants and splicing events (see Fig. 1).

Based on sequence alignment result, ISVASE first filters mapped reads using stringent rule-dependent filters, such as low base quality (<Q30), low mapping quality (unpaired reads for paired-end data, PCR duplication, quality control, multiple mapping, mismatch, insertion and deletion) and short read length (<30 bp). Only split-reads with at least 8 bp anchor size in both parts of splicing event (junction) can be used to identify putative splicing event. Initially, splicing events with low read depth (<3) are discarded. Low abundant splicing events are also filtered out as background expression by applying Fisher’s exact test to the putative splicing event and its related splicing events (sharing 5’ss or 3’ss). Here, ISVASE divides each splicing event into two independent parts based on 5’ss and 3’ss. ISVASE can remove known splicing events using annotation file in GTF, GFF or BED format by option “-k no”. Although excellent software for sequence variant calling has existed such as GATK [14] and samtools [15], their results are hard to be used for SVASE calling, which needs to clarify specific sequence variants for unique splicing event. Thus, ISVASE adopts de novo sequence variant identification by only using junction-supporting split-reads. The observed sequence variant candidates are filtered by following criteria: reads depth (<3), alternative allele (ALT) supporting reads number (<3), ALT proportion (<0.1) and the significance of variant (*p* > 0.05, Fisher’s exact test).

The practice of SVASE identification has a bit difference depending on whether the ALT frequencies are consistent between target splicing event and all related splicing events. We calculated the ALT frequencies for each sequence variants using reads of all splicing events and the target splicing event, respectively. If consistence, the association is assessed only using reads from target splicing event. Otherwise, total related reads are used. ISVASE applies same method as PVAAS to evaluate the significance of association. Besides, ISVASE assesses splicing signal by MaxEntScan [16] and identifies junction shift events to reduce the false positive of splicing event calling. Furthermore, DNA mutation and/or RNA editing profiles (like dbSNP [17], DARNED [18], RADAR [19] or user provided DNA mutation or RNA editing sites) can be used to assign the source type of sequence variants. ISVASE outputs the detailed statistical results with figures and tables. ISVASE also extracts the flanking sequence for sequence variants, which can be used to predict exonic splicing enhancer (ESE) motifs using tools like ESEfinder [20] and Human Splicing Finder [21]. The output of identified SVASEs can be accepted by ANNOVAR [22] and SnpEff [23] for further functional analysis like KEGG pathway and Gene Ontology. The code of ISVASE was written using Perl (v5.18.4), the figures were created by R (v3.1.2) while the sequence alignment file was operated by samtools (v1.2).

## Results and Discussion

To demonstrate the functionality of ISVASE and compare with PVAAS, PVAAS testing data (downloaded from website http://pvaas.sourceforge.net/) was used. PVAAS (v0.1.5) identified 8 SVASEs (belonging to new splicing events), while ISVASE obtained 172 SVASEs and 14 of them were new splicing events (Table 1, Additional files 1 and 2). Two software only share one SVASE, which probably is genuine according to dbscSNV [24]. Among other 7 PVAAS unique SVASEs, 1 SVASE has a low ALT ratio (<=0.01), 1 SVASE is supported by un-split reads and remaining 5 SVASEs are identified only by a small part of target junction supporting reads (0.8% ~8%). All of these error-prone SVASEs have been filtered in our tool. All 14 SVASEs belonging to new splicing events in our result have high confident evidences such as mapping quality, ALT reads and other filter criteria mentioned above. Among 158 SVASEs in known splicing events, 55 SVASEs are non-reference homozygous, 66 SVASEs have more than 80% ALT reads, and 110 SVASEs have more than 50% ALT reads. Comparing SVASEs with dbSNP and RADAR database, we found that ISVASE has better performance than PVAAS both for novel and all SVASEs (Table 2). Moreover, ISVASE run faster than PVAAS. For test data (7.26 million reads), PVAAS takes 1.63 h, while ISVASE only needs 11 min for novel splicing events or 13 min for all splicing events (Table 3).

**Table 1:** The statistics of SVASE identification using PVAAS and ISVASE

Data	PVAAS			ISVASE(novel)			ISVASE(all)		
	Total	dbSNP	RADAR	Total	dbSNP	RADAR	Total	dbSNP	RADAR
PVAAS test data	8	0	0	14	7	0	172	129	0
Control1(SRR388226)	61	12	0	134	54	1	2577	2138	3
Control2(SRR388227)	63	9	0	120	50	2	2557	2130	3
Control(common)	28	2	0	87	36	1	2105	1788	2
Knockdown1(SRR388228)	93	18	0	187	83	1	2710	2250	2
Knockdown2(SRR388229)	89	24	0	168	73	1	2760	2293	1
Knockdown(common)	31	8	0	119	55	1	2298	1951	1

**Table 2:** The performance comparison between PVAAS and ISVASE

Data	Method	Precision	Consistency
PVAAS test data	PVAAS	0.00(0/8)	-
	ISVASE(novel)	0.50(7/14)	-
	ISVASE(all)	0.75(129/172)	-
Control1(SRR388226)	PVAAS	0.20(12/61)	0.46(28/61)
	ISVASE(novel)	0.40(54/134)	0.65(87/134)
	ISVASE(all)	0.83(2138/2577)	0.82(2105/2577)
Control1(SRR388227)	PVAAS	0.14(9/63)	0.44(28/63)
	ISVASE(novel)	0.42(50/120)	0.73(87/120)
	ISVASE(all)	0.83(2130/2557)	0.82(2105/2557)
	PVAAS	0.07(2/28)	-
Control(common)	ISVASE(novel)	0.41(36/87)	-
	ISVASE(all)	0.85(1788/2105)	-
Knockdown1(SRR388228)	PVAAS	0.19(18/93)	0.33(31/93)
	ISVASE(novel)	0.44(83/187)	0.64(119/187)
	ISVASE(all)	0.83(2250/2710)	0.85(2298/2710)
Knockdown2(SRR388229)	PVAAS	0.27(24/89)	0.35(31/89)
	ISVASE(novel)	0.43(73/168)	0.71(119/168)
	ISVASE(all)	0.83(2293/2760)	0.83(2298/2760)
Knockdown(common)	PVAAS	0.26(8/31)	-
	ISVASE(novel)	0.46(55/119)	-
	ISVASE(all)	0.85(1951/2298)	-

*Precision* known SVASE/total SVASE, known SVASE defined as SVASE existed in dbSNP, *Consistency* common SVASE/total SVASE, common SVASE means the SVASE identified in both repeat samples

**Table 3:** The running time comparison between PVAAS and ISVASE

Data	PVAAS	ISVASE(novel)	ISVASE (all)
PVAAS test data	1h38m25s	11m22s	13m11s
Control1(SRR388226)	12h5m22s	2h27m31s	2h52m33s
Control2(SRR388227)	12h52m19s	2h29m50s	2h53m17s
Knockdown1(SRR388228)	15h45m40s	2h37m36s	3h4m3s
Knockdown2(SRR388229)	16h40m40s	2h42m27s	3h9m38s

To further reveal the advantage of ISVASE, we also test another real data set with 4 RNA-seq samples for human glioblastoma cell line U87MG (SRR388226 and SRR388227 are control samples and SRR388228 and SRR388229 are *ADAR* knockdown samples) [25]. The raw data was trimmed by Trimmomatic [26] and aligned by GSNAP (only concordant mapping results were used for downstream analysis) [12]. Using ISVASE, 134 and 120 SVASEs (87 common) were obtained for control data, while 187 and 168 SVASEs (119 common) for knockdown data in new splicing events. If considering all splicing events, 2105 and 2298 common SVASEs were identified in control and knockdown data (Table 1, Additional files 3, 4, 5, and 6). In each sample, at most three SVASEs belonging to RNA editing sites in RADAR database were detected (totally four SVASEs belonging to RADAR database), and more than 82% SVASEs existed in dbSNP. In comparison, PVAAS got 61 and 63 SVASEs (28 common) for control data, while 93 and 89 SVASEs (31 common) for knockdown data (Table 1, Additional files 7, 8, 9, and 10). In PVAAS result, there wasn’t any SVASE belonging to RNA editing sites in RADAR database and at most 27% SVASEs existed in dbSNP. These results indicated that PVAAS has higher false positive rate comparing with ISVASE (Table 2). Using repeat samples, we also found that PVAAS has lower consistency rate comparing with ISVASE (about 47% vs. about 83%) (Table 2). Moreover, for each sample, ISVASE showed an advantage of running time to PVAAS (about 3 h vs. 14.34 h) (Table 3).

**Fig. 2:**
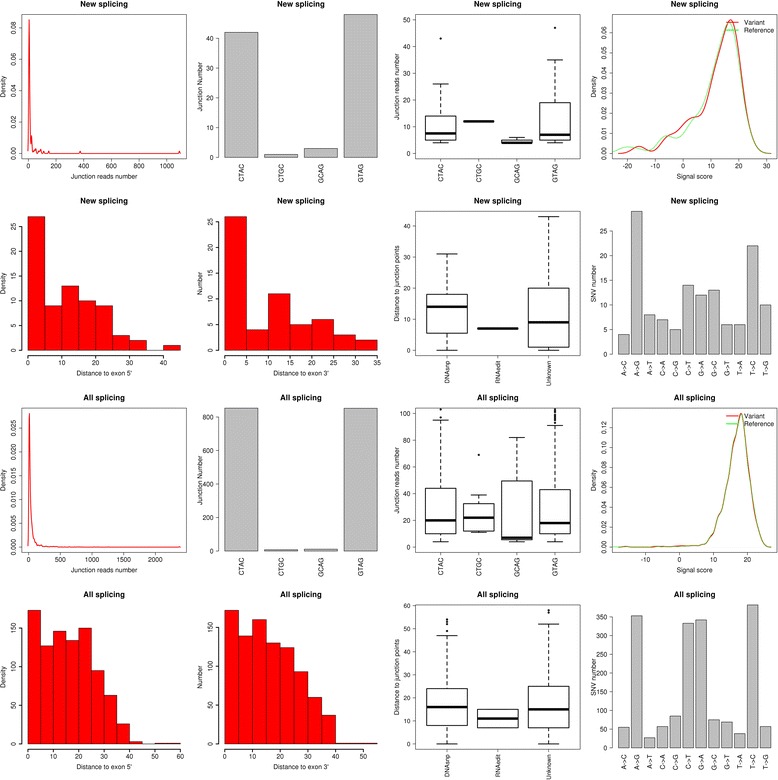
The characteristics of SVASEs between novel and all SVASE sites in sample SRR388226. The density of junction reads number, the bar plot of junction number for different junction splicing signals, the boxplot of junction reads number distribution for different junction splicing signals, the density of splicing signal score for variant replaced sequence and reference sequence, the histogram plot of distances between sequence variant and exon 5′ side, the histogram plot of distances between sequence variant and exon 3′ side, the boxplot of distance distribution between sequence variant type and junction breakpoint, and the bar plot of sequence variant number for different sequence variant types are shown for SVASEs located in new splicing events (*the upper half*) and all splicing events (*the lower half*)

The SVASEs identified by ISVASE can be used for downstream analysis easily. For example, we used 65 common SVASEs in new splicing events from the above four samples to do further analysis. We annotated these SVASEs by ANNOVAR and found 28 related genes (Additional file 11). Among them, 20, 9 and 8 SVASEs located in *HLA*, *HCG4B* and *AHNAK2* genes. *HLA* genes play important roles in tumor immune surveillance and escape, and *HCG4B* gene is a pseudogene of HLA complex group. *AHNAK2* gene is associated with calcium channel proteins and its exon 7 size is almost 18 kb. We found 8 SVASEs associated with 5 new splicing events inside the exon 7. Gene Ontology enrichment analysis found these 28 genes are significantly enriched in cancer related functions, such as antigen processing and presentation, response to type I interferon and interferon-gamma (Table 4). We also used ESEfinder to detect ESE motifs and found 57 of 65 SVASEs located in predicted ESE motifs. This result indicates most of SVASEs perform their function possibly by influencing ESE motifs of splicing events. Moreover, SVASEs have some basic characteristics (using SRR388226 data as an example), such as high proportion of canonical splicing signal GT-AG (or reverse complement CT-AC), similar signal scores for splice sites with reference or alternative allele, tendency to junction breakpoints, and high frequency of A- > G/T- > C and G- > A/C- > T transitions (58.96% in new splicing events and 75.13% in all splicing events) (Fig. 2).

**Table 4:** Gene Ontology enrichment analysis for genes related with 65 common SVASEs using PANTHER (filtered redundant records)

GO function	Total gene	SVASE gene	Expected	Fold Enrichment	*P* value (<0.05)
GO biological process complete					
antigen processing and presentation of endogenous peptide antigen via MHC class I	15	3	0.02	>100	0.00541
antigen processing and presentation of peptide antigen via MHC class I	108	6	0.12	50.28	1.51E-05
antigen processing and presentation of endogenous antigen	19	3	0.02	>100	0.011
antigen processing and presentation of exogenous antigen	181	6	0.2	30	0.000317
response to type I interferon	74	6	0.08	73.37	1.6E-06
response to interferon-gamma	151	6	0.17	35.96	0.000109
GO molecular function complete					
antigen binding	107	6	0.12	50.75	4.69E-06
GO cellular component complete					
MHC protein complex	30	6	0.03	>100	1.14E-09
membrane-bounded vesicle	1169	9	1.29	6.97	0.00285
vesicle membrane	508	7	0.56	12.47	0.00116

## Conclusions

ISVASE provides users to identify SVASEs simply and fast using RNA-seq data. It identifies SVASEs for both parts of splicing event (or junction) separately. To reduce false positives due to sequencing errors, ISVASE applies several stringent rule-depended filters and statistical filters in different steps. ISVASE can evaluate junction shift events and junction signals (5′ ss and 3′ ss) to remove false positive splicing events. It also can use user provided DNA mutation and/or RNA editing data to designate types of sequence variants. To facilitate downstream analysis, ISVASE obtains flanking sequences and VCF output for other tools usage. ISVASE also provides 6 tables and 8 figures to describe the characteristics of SVASEs. In summary, our approach enabled de novo identification of SVASEs, which sets the stage for further mechanistic studies.

## Additional files

Additional file 1: PVAAS result for its test data. (XLS 753 bytes)

Additional file 2: ISVASE result for PVAAS test data. (XLS 17 kb)

Additional file 3: ISVASE result for SRR388226. (XLS 365 kb)

Additional file 4: ISVASE result for SRR388227. (XLS 362 kb)

Additional file 5: ISVASE result for SRR388228. (XLS 384 kb)

Additional file 6: ISVASE result for SRR388229. (XLS 391 kb)

Additional file 7: PVAAS result for SRR388226. (XLS 4 kb)

Additional file 8: PVAAS result for SRR388227. (XLS 4 kb)

Additional file 9: PVAAS result for SRR388228. (XLS 6 kb)

Additional file 10: PVAAS result for SRR388229. (XLS 6 kb)

Additional file 11: Genes of 65 common SVASEs in new splicing events identified by ISVASE for four samples. (DOCX 12 kb)

## Abbreviations

3′ ss: 3′ splice site
5′ ss: 5′ splice site
ALT: alternative allele
ESE: exonic splicing enhancer
ISVASE: Identification of sequence variant associated with splicing event
PVAAS: Program to identify variants associated with aberrant splicing
SE: splicing event
SV: Sequence variant
SVASE: Sequence variant associated with splicing event
