# Supplementary material for: ISVASE: identification of sequence variant associated with splicing event using RNA-seq data
Source: BMC Bioinformatics. 2017 Jun 28;18:320. doi: 10.1186/s12859-017-1732-7 (PMC5490186; doi:10.1186/s12859-017-1732-7)
Supplement: Supplementary file 11 — Genes of 65 common SVASEs in new splicing events identified by ISVASE for four samples. (DOCX 12 kb) [file 12859_2017_1732_MOESM11_ESM.docx]

**Additional file 11.** Genes of 65 common SVASEs in new SEs identified by ISVASE for four samples

| **Gene** | **SVASE No.** | **Gene** | **SVASE No.** | **Gene** | **SVASE No.** |
| --- | --- | --- | --- | --- | --- |
| *HLA-C* | 4 | *SDF4* | 1 | *LOC101927592* | 2 |
| *PLAC8* | 1 | *TP53TG3* | 1 | *LINC01452* | 1 |
| *DNER* | 2 | *AHNAK2* | 8 | *PROS1* | 1 |
| *SLC47A1* | 1 | *HLA-B* | 3 | *NFYB* | 2 |
| *HLA-A* | 7 | *ACACA* | 2 | *LOC100132352* | 1 |
| *PRRC2C* | 1 | *NT5C2* | 1 | *CFAP45* | 1 |
| *HLA-G* | 2 | *HCG4B* | 9 | *XPO7* | 1 |
| *SORBS2* | 1 | *LMO7* | 1 | *HLA-H* | 3 |
| *LCORL* | 3 | *GABRG3* | 1 |  |  |
| *KIAA0825* | 3 | *HLA-F* | 1 |  |  |
